# Willingness to accept metaverse safety training for construction workers based on extended UTAUT

**DOI:** 10.3389/fpubh.2023.1294203

**Published:** 2024-01-05

**Authors:** Zhenxu Guo, Qinge Wang, Chunyan Peng, Sunning Zhuang, Biao Yang

**Affiliations:** ^1^School of Civil Engineering, Central South University, Changsha, China; ^2^Guangzhou Expressway Co., LTD., Guangdong, China

**Keywords:** metaverse, safety training, construction worker, unified theory of acceptance and use of technology, structural equation modeling

## Abstract

Safety training (ST) is essential in avoiding unsafe behavior of construction workers. With the rise of metaverse technology, metaverse safety training (MST) has gradually become a new model to guide construction workers in safety production. An in-depth study of construction workers’ willingness to accept the metaverse safety training (WAMST) helps improve its effectiveness, but studies need to pay more attention to it. This study constructs a conceptual model of WAMST for construction workers, and the influencing factors of WAMST are explained based on the extended Unified Theory of Acceptance and Use of Technology (UTAUT). It established a Structural equation modeling to verify the relationship between influencing factors. An example verifies the feasibility of the model. The results show that the framework significantly contributes to the willingness of construction workers to participate and improves safety awareness. Specifically, performance expectancy, effort expectancy, social influence, and convenient conditions significantly affect the construction workers’ willingness to accept. Convenient conditions have a direct effect on actual behavior. Willingness to accept plays a mediating role between performance expectancy and actual behavior. Perceived trust moderates the effect between willingness to accept and actual behavior, and the force of positive interpretation increases proportionally. It confirms how to improve the safety capacity of construction workers and provides references for governments, enterprises, and projects to formulate ST strategies.

## 1 Introduction

Safety training (ST) for construction workers represents a topic of intense interest for policymakers and academics alike (1, 2). In order to fully demonstrate the characteristics, advantages, and disadvantages of safety training in different countries and cultural backgrounds, Singapore, China and the United Kingdom are representative. The Singapore government stipulates that construction workers must undergo safety training, which lasts one day. China has established a three-level safety education system. The Safety and Health at Work requires all construction workers in the UK to take safety courses for at least eight hours. Indeed, ST is acknowledged as an effective way of avoiding potential risks and taking appropriate action, representing critical preventive measures (3, 4).

However, the issues associated with the construction workers’ ST, such as single training content, outdated training methods, and lack of continuity, necessitate a revolutionary approach (5–8). Recent advances in digital twins, augmented reality, virtual reality, and artificial intelligence have created avenues to transform the construction workers’ ST. (9–12) In this regard, one perspective solution is using metaverse to carry out immersive and interactive ST. (13, 14) There have been some efforts aiming to provide metaverse safety training (MST). MST is defined as an innovative way of ST that is usually performed as absolute, repeatable, targeted, interactive, high security, cost-saving, and immersive (15–18). It is advantageous to enter a virtual security education scenario to feel the application of relevant safety knowledge (19–21). This immersive simulation experience can make construction workers aware of the importance of safety issues and increase their attention to ST. Notably, MST has shown great potential to improve the safety awareness and skill level of construction workers (22). Given its prominent advantages, these advantages cannot be maximized if users do not adopt it. MST is in the initial stage. Thus, determining the factors affecting the construction workers’ willingness to accept the metaverse safety training (WAMST) is still in short supply.

To that end, scholars in technology adoption have used various methods to identify the construction workers’ willingness to use technology, such as the Unified Theory of Acceptance and Use of Technology (UTAUT), and technology acceptance model (23). Compared to other methods, research indicated that the UTAUT provides a better understanding of the variance in the behavioral intention to use a technology (24, 25). UTAUT integrates the technology acceptance model, planned behavior theory, and social cognition theory, which scholars use to discuss the willingness to accept (WA) for a sure thing or behavior (26–28). It provides a basic framework for interpreting and predicting the WA. Therefore, this study explores the factors affecting WAMST for construction workers with the help of UTAUT. Specifically, the knowledge gaps include: (1) What factors affect the construction workers’ WAMST? (2) How do these factors affect the construction workers’ WAMST? (3) What is the relationship among these factors?

The structure of this study is organized as follows. We discuss related literature reviews and hypotheses in section 2. Section 3 interprets the research methodologies. In section 4, this study identifies factors and paths and then discusses the relationship among factors in section 5. Finally, Section 6 concludes this study.

## 2 Literature reviews and hypotheses

### 2.1 Safety training for construction workers

ST for construction workers is the key to the construction enterprises achieving safety management (29). It is intended to exploit various training methods to accomplish systematic and compulsory tasks in construction sites. However, ST for construction workers is poor performance, triggering complications. Such complications are affected by numerous factors, including risk-taking behavior attitude, risk perception, unsafe construction equipment, geological exploration design, technical management, safety management, and construction site conditions (30–32). In this regard, many studies have suggested that chatbots and virtual training platforms can enhance the training effect (4, 33). The new technology promotes the updating and upgrading training content, and ST based on virtual reality plays an increasingly important role.

Traditional ST for construction workers is mainly offered through centralized training, explanation on the spot, and video teaching. Training content usually includes safety regulations, safety operation procedures, and emergency treatment methods. While such ST can improve construction workers’ emergency response ability and cover multiple workers, it is not capable of providing an overall interactive, hands-on experience. Simulation drill is another method construction workers can learn through hands-on sessions. However, it is often criticized for being costly and time-intensive. In this vein, a metaverse that can provide repeat drills, interactive learning, and experience is a viable option (34). The metaverse is an ecosystem built by integrating digital twins, augmented reality, virtual reality, artificial intelligence, and other technologies (20). Jeremy Bailenson of Stanford University sees education as the “killer app” of the metaverse for the foreseeable future. Similarly, Professor Zhu Jiaming, an economist, has pointed out that the most extensive potential area for metaverse applications is education and training.

MST is to break the constraints of conditions through the metaverse so that participants can feel the scary scene in their place and further understand the content of operation points, risk levels, and emergency disposal more deeply (13). This model has become a revolutionary innovation in the field of ST. Video, animation, and other carrier forms enrich the digital education and training resources, will be hard hat impact, high-risk scenes such as falling a complete presentation, improve the safety awareness of construction workers (35). Compared with traditional ST, it effectively avoids problems such as difficulty understanding written language, poor image of safety manuals, and boring meetings. Immersive training can make construction workers remember more profoundly and effectively reduce the occurrence of safety accidents (36). However, as a new model, MST is often accompanied by instability. It also affects the WAMST, which negatively affects the safety of construction workers (37).

To sum up, the metaverse is just beginning, and the application of MST in the architecture, construction, and engineering industry still needs to be deeply explored. Combined with the characteristics of metaverse, the study of WAMST is helpful to understand the advantages and limitations of MST and provide guidance for optimizing the design and implementation. Therefore, this study attempts to explore the influencing factors of the WAMST, reveal the influence mechanism of MST, and deepen the micro-interpretation of the metaverse.

### 2.2 Theoretical hypothesis

In order to select an appropriate model covering almost all factors affecting the construction workers’ WAMST, the UTAUT is dedicated as the theoretical basis to propose the conceptual model in the study. Generally speaking, performance expectancy (PE), effort expectancy (EE), social influence (SI), and convenient conditions (CC) are the four variables affecting the WA, and CC can directly predict the possibility of actual behavior (AB). According to the research, perceived trust (PT) is found as the crucial factors. In that, this study illustrates that the construction workers’ WAMST is affected by PE, EE, CC, SI, WA, and PT.

Affected by the characteristics of construction workers, construction workers need better safety awareness and even resist traditional and boring ST. MST allows construction workers to personalize learning and invest less time. Compared with the traditional ST, it realizes the digitalization of training management so that construction workers can effectively realize self-improvement and meet their work and learning needs (10). Companies and projects reward and punish construction workers according to their performance of MST to motivate them to improve their safety performance to a certain extent (38). As a new model, MST satisfies the exploration desire of construction workers. In conclusion, when the perceived usefulness of construction workers for MST is high, perceived usefulness acts on PE and makes construction workers more active for WAMST.

EE manifests in the perceived ease of use of construction workers conducting MST. MST shows the actual construction site environment through an auditory and visual simulation. In dangerous situations, construction workers can intuitively feel, interact, immerse in the scene, and generate a positive attitude towards ST. (39) Construction workers are more satisfied with this exciting training method and are more willing to participate.

SI comes from the government’s intention to strengthen the standardized construction of construction crews. Under the guidance of the government policy, whether it is the company, the project, or the team, the supervision of the construction workers to participate in MST will increase, forcing the construction workers to accept a new training mode. Through the deep integration of digital technology and safety knowledge, the multi-level sensory experience can improve the willingness of construction workers to participate in ST. In addition, the cognition of construction workers to safety and the interaction between colleagues significantly impact the safety atmosphere, which further affects the WAMST (40).

CC is the outstanding advantage of MST, which differs from traditional ST. The concrete manifestation is environmental convenience, economic convenience, knowledge convenience, and technological convenience. MST enables construction workers to conduct ST anytime and anywhere without time and space constraints (21). Construction workers need no additional investment, can use their smartphones to attend the training, and do not have to worry about traffic usage. Almost all projects provide free internet services to ST for construction workers, such as mobile phone downtime, excessive phone charges, and other situations. Metaverse technology imparts strong theoretical learning principles in ST, which is more intuitive in understanding safety knowledge (41). Intelligent sense, active service, and other methods reduce the difficulty of learning and operation (42). It not only enhances the WAMST for construction workers but also enhances the training effect.

Based on the above analysis, the following hypotheses are proposed:

*H1*: Performance expectancy has a positive effect on willingness to accept.

*H2*: Effort expectancy has a positive effect on willingness to accept.

*H3*: Social influence has a positive effect on willingness to accept.

*H4*: Convenient conditions has a positive effect on willingness to accept.

*H5*: Convenient conditions has a positive effect on actual behavior.

In the early application stage, new technology will be widely questioned, including professionalism, authenticity, advanced, systematic, privacy, and service. Suppose the construction workers have a higher degree of trust in MST. In that case, the construction workers will better follow the safety rules, training performance, and safety beliefs (43). Given the substantial data perception, collection, analysis, and personalized recommendation capabilities of the metaverse technology, construction workers are particularly concerned about personal privacy leakage. The metaverse can compensate for the difference in safety perception between construction workers and managers by making targeted measures according to different team attributes.

The WA directly affects the AB for construction workers to participate in MST. The WA is divided into three levels: their intention, recommend others, and suggest promotion. The stronger the WA for construction workers, the higher the training frequency, duration, and persistence. When the WA is at a certain level, the higher the PT, the more positive the AB will be. In other words, PT significantly moderates the relationship between WA and AB (44).

Based on the above analysis, the following hypotheses are proposed:

*H6*: Willingness to accept has a positive effect on actual behavior.

*H7*: Perceived trust moderates the positive effect of willingness to accept on actual behavior.

**Figure 1:**
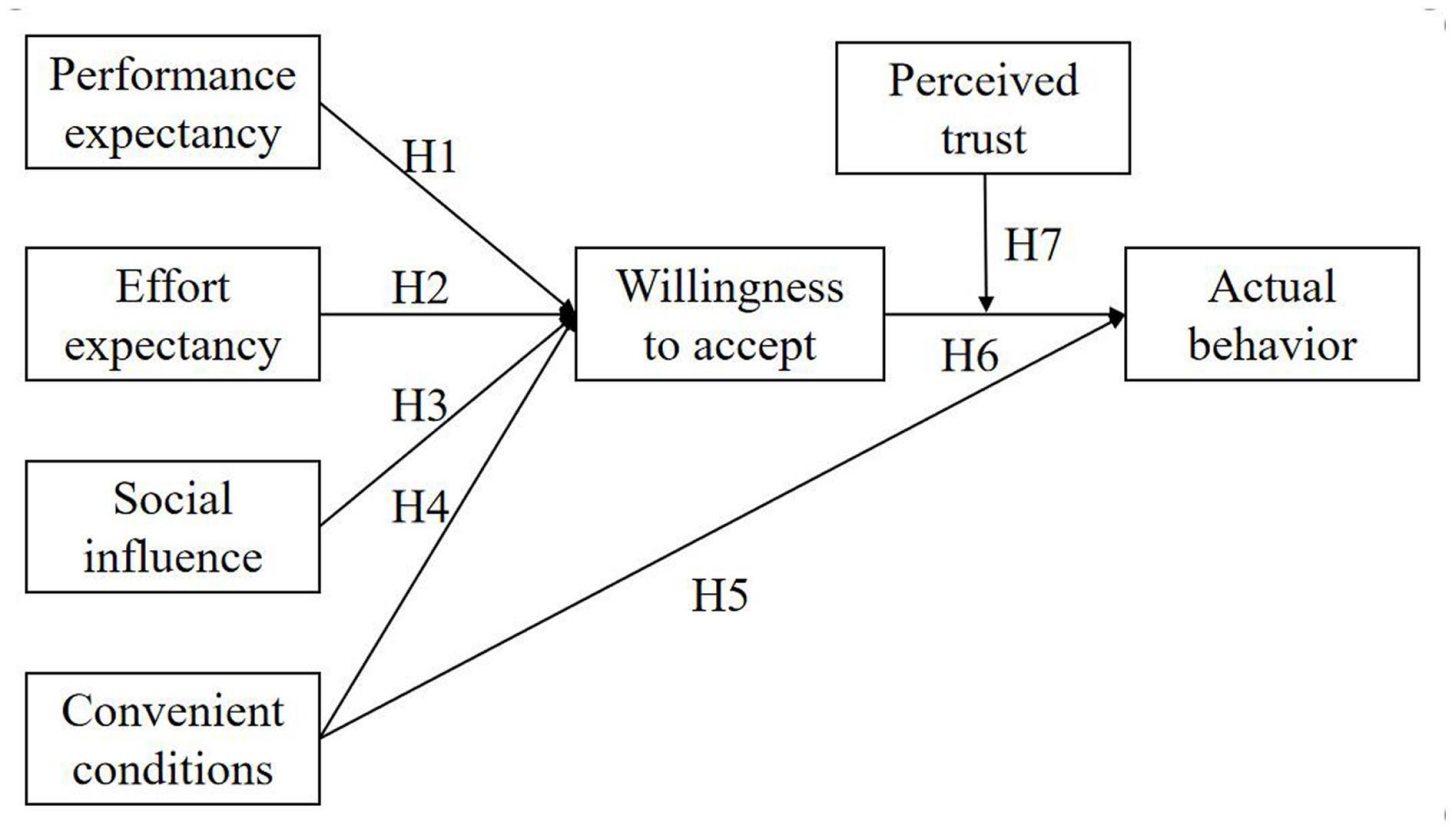
The extension of UTAUT.

To sum up, Figure 1 shows the proposed conceptual model.

## 3 Methodology

### 3.1 Research flowchart

**Figure 2:**
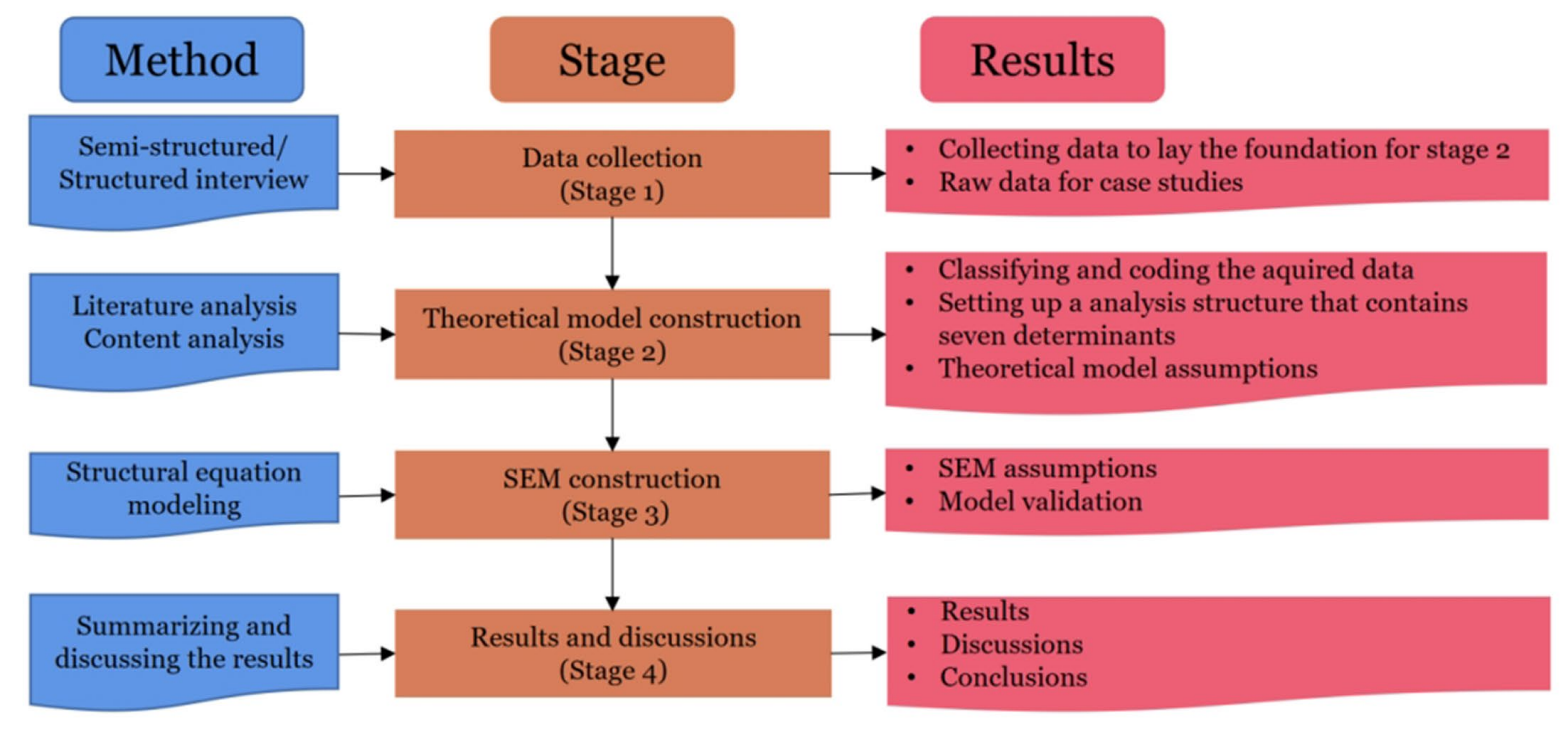
Research flowchart.

This study adopted a mixed research method, including four main stages (see Figure 2).

First, this study conducted a group survey in Hunan Province, China, covering Changsha, Zhuzhou, Xiangtan, Yueyang, Changde, and other cities. From March 1 to March 6, 2023, the first field survey was carried out to analyze the characteristics of MST, such as the blending of virtual reality, immersion experience, and multiple interactions, to clarify the current situation of interaction, feedback, and incentive of ST for construction workers. One-on-one chat interviews were conducted to guide interviewees to answer questions related to MST based on their knowledge and experience. The interviewees have some knowledge and exposure to MST, covering different age and gender groups. It helps to ensure the reliability and validity of the study and avoid data bias caused by individual heterogeneity. They are from three teams engaged in road, bridge, and tunnel engineering. Each team interviewed seven people, a total of 21 construction workers. Each person was interviewed for 10–30 min, and the whole interview process was recorded and, on this basis, combined with the relevant literature preliminary design of the survey questionnaire.

Next, the research group summarized the collected data, which became the foundation of the theoretical model. Based on the characteristics of MST and combined with the practice of ST for construction workers, this study establishes the conceptual framework of WAMST. The framework includes PE, EE, SI, CC, PT, WA, and AB. The factors of each dimension, source references, and source cases are shown in Table 1. On March 9, the research group invited three experts from Central South University and Shenzhen Zhonghan Technology Development Co., Ltd. to discuss the scientificity and measurability of the questionnaire indicators to form a preliminary questionnaire. On March 10, the research group conducted the second field survey and organized the Yiyang-Changde Expressway expansion project construction workers to pre-answer the questionnaire. The pilot study was conducted to verify the appropriateness and completeness of influencing factors. According to the interview results, group members discussed in groups to get the final questionnaire.

**Table 1:** Measurement items and survey questionnaires.

Constructs	Measurement items	1	2	3	4	5	6	7	8	9
Performance expectancy (PE)	PE1 Reduce input	✔	✔	✔	✔	✔	✔	✔	✔	✔
	PE2 Get rewards									
	PE3 Improve oneself									
Effort expectancy (EE)	EE1 Sensory stimulation		✔		✔	✔	✔	✔	✔	✔
	EE2 Virtual interaction									
	EE3 Immersion experience									
	EE4 Perceived pleasure									
Social influence (SI)	SI1 Policy guidance	✔	✔	✔	✔	✔	✔	✔	✔	✔
	SI2 Project progress									
	SI3 Other’s influence									
	SI4 Individual innovation									
Convenient conditions (CC)	CC1 Environmental convenience	✔	✔	✔					✔	✔
	CC2 Economical convenience									
	CC3 Intellectual convenience									
	CC4 Technical convenience									
Perceived trust (PT)	PT1 Professionalism	✔			✔			✔		✔
	PT2 Authenticity									
	PT3 Advancement									
	PT4 System									
	PT5 Privacy									
	PT6 Service									
Willingness to accept (WA)	WA1 Suit oneself	✔	✔		✔		✔		✔	
	WA2 Recommend others									
	WA3 Suggest promotion									
Actual behavior (AB)	AB1 Useful frequency	✔	✔	✔	✔	✔	✔	✔	✔	
	AB2 Service time									
	AB3 Continuous use intention									

1— (45); 2— (46); 3— (47); 4— (48); 5—- (49); 6— (50); 7— (51); 8— (52); 9— (41).

Then, after the pilot study, in April 2023, the research group distributed questionnaires to the projects that adopted the MST in Hunan Province through face-to-face interviews, Tencent conference discussion, questionnaire star survey, and other methods. According to the questionnaire results, we conducted the descriptive statistics on sample data and the data reliability and validity test. As well, the structural equation modeling (SEM) of the influencing factors of WAMST is constructed. SEM, as a multivariate statistical method, includes the model preparation stage and the model test stage. In the model preparation stage, SEM is constructed according to the practical and series of theoretical research on the ST of construction workers. In the model test stage, AMOS software was used to test and verify the model’s validity. The model includes seven latent variables and 27 manifest variables. Among them, PE, EE, SI, and CC are prerequisite variables, WA is a mediating variable, PT is a moderating variable, and AB is the outcome variable.

Finally, we summarized and discussed the analysis results, and the theoretical and practical implications were proposed.

### 3.2 Data collection and analysis

This study investigate some projects, including Sangzhi-Longshan Expressway, Xining-Xinhua Expressway, Chaling-Changning Expressway, Liling-Loudi Expressway, and the Yiyang-Changde Expressway expansion project. Considering the characteristics and professional nature of construction workers, this study also explained factors to ensure the accuracy of understanding. For example, “sensory stimulation” refers to the degree of visual and auditory stimulation brought to construction workers by the quality of video animation and sound effects shown in MST. “Virtual interaction” refers to the possibility of solid substituting virtual scenes to attract construction workers to carry out active interaction. “Immersive experience” refers to the satisfaction of construction workers with this immersive ST. “Perceived pleasure” refers to the fun of MST as a new training mode and its positive effect on the ST of construction workers. The questionnaire consists of two parts.

(1) Basic information of interviewees (education background, work experience, project type, and identity characteristics)
(2) Respondents’ attitudes towards the importance of 27 influencing factors were measured by Richter’s five-level scale (1 = very important, 2 = not important, 3 = average, 4 = important, 5 = very important).

For 353 responses, the research group excluded the questionnaire that time spent filling was less than 60 s and the same item score. There are 310 valid questionnaires, and the effective rate of the questionnaire is 87.82%. The number of valid questionnaires was more than 10 times the entire item, and the samples met the criteria for statistical analysis. SPSS software was used for statistical analysis of the questionnaire data, as shown in Table 2.

**Table 2:** General information of the respondents.

Respondents	Categorization	Number	Percentage
Project types	Road engineering	118	38.06%
	Bridge engineering	101	32.58%
	Tunnel engineering	61	19.68%
	Others	30	9.68%
Related work experience	1–3 years	106	34.19%
	4-7 years	140	45.16%
	>8 years	64	20.65%
Education background	Primary school degree	21	6.77%
	Junior high school degree	115	37.10%
	Senior high school degree	97	31.29%
	College degree and above	77	24.84%
Whether he is the team leader	Yes	108	34.84%
	No	202	65.16%
Whether he is special worker	Yes	49	15.81%
	No	261	84.19%

As can be seen from Table 2, the proportion of construction workers who participated in the questionnaire survey engaged in road engineering, bridge engineering, and tunnel engineering was similar and evenly distributed in the five highway projects. Although the education level of construction workers is generally low, 65.81% of construction workers have engaged in related work for more than 4 years. The rich work experience gives construction workers a deeper understanding of the MST, further ensuring data quality. The survey objects cover team leaders and special operators. The duty characteristics of team leaders and the work characteristics of special operators make them different from general construction workers in WAMST, which makes the questionnaire data more comprehensive.

## 4 Results

### 4.1 Descriptive statistics on sample data

Descriptive statistics analyzed the data distribution of observed variables, including the mean value, standard deviation, skewness, and peak value of the sample. Among the 310 questionnaires, respondents’ scores on the items were all between 1 and 5, with the mean [3.20, 4.20] and standard deviation [0.759, 1.068]. The overall distribution was reasonable. In the test of normal distribution of data, the absolute values of skewness coefficients and kurtosis coefficients of data obtained by all observed variables are less than three and less than eight, conforming to normal distribution. Therefore, the maximum likelihood estimation method is used to analyze the SEM.

### 4.2 Data reliability and validity test

Cronbach’s α coefficient was used to test the scale’s internal consistency. According to the questionnaire reliability and validity test results, the corrected item-total correlation of all items was more significant than 0.3, and Cronbach’s α coefficient of the scale as a whole was 0.892, indicating that the questionnaire has internal consistency. The Kaiser-Meyer-Olkin and Bartlett sphericity test results showed that the KMO value was 0.855 < 0.6, and *p* < 0.001 indicates that scale data are suitable for factor analysis. SPSS was further used for factor analysis, and the results showed that the cumulative variance interpretation rate of seven factors was 70.341%, which was greater than 50%, indicating that these seven factors could effectively extract the item information.

### 4.3 Structural equation modeling test

**Figure 3:**
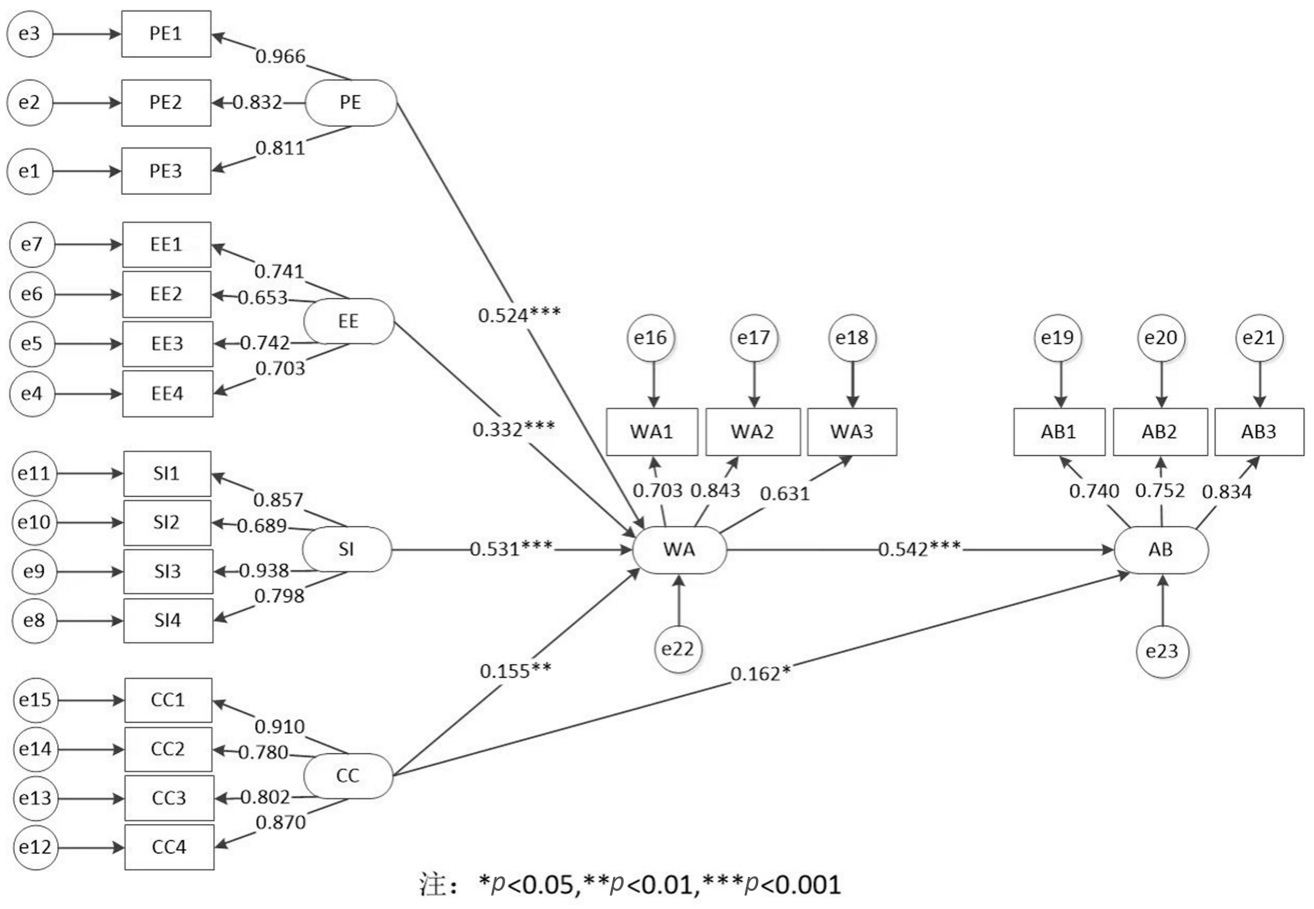
SEM results.

This study constructed an SEM to investigate further the direct influence of the measured variables in the conceptual model shown in Figure 1. The results showed that all fitting indexes of the model were good (*X*^2^/df = 5.110, GFI = 0.973, CFI = 0.931, NFI = 0.901, RMSEA = 0.015), indicating that the constructed conceptual model was good. According to the theoretical model and relational hypothesis, the sample data was imported into Amos 22.0, and the standardized path coefficients of the model were obtained through analysis, as shown in Figure 3. From the interpretation rate of the overall model, the square R^2^ value of the multivariate correlation coefficient of construction workers’ WAMTP is 0.690, indicating that the variation of the WA by all external variables is 69.0%, and the explanatory force of variables is strong. According to the path coefficient and influence significance between variables, all hypotheses passed the hypothesis test, and the direct effect hypothesis was verified. The standardized path coefficient of the direct effect of PE on WA is 0.524, *p* < 0.001, C.R. was 8.467, and the absolute value was more significant than 1.96, indicating that PE had a statistically significant positive effect on WA. Therefore, the original hypothesis was accepted, and H1 was established. The same goes for H2, H3, and H6. The standardized path coefficient of the direct effect of CC on WA is 0.155, *p* = 0.002 < 0.05, C.R. was 3.049, and the absolute value was more significant than 1.96, indicating that CC had a statistically significant positive effect on WA. Therefore, the original hypothesis was accepted, and H4 was valid. The standardized path coefficient of the direct effect of CC on AB is 0.162, *p* = 0.010 < 0.10, C.R. was 2.581, and the absolute value was more significant than 1.96, indicating that CC had a statistically significant positive effect on AB. Therefore, the null hypothesis was accepted, and H5 was valid.

As can be seen from Figure 3, the factor loads of each latent variable affecting the observed variable are all positive, indicating a statistically significant influence. The factor loads of the three observed variables of PE were 0.966, 0.832, and 0.811, respectively. The reliability of the measurement model was good. As for the standardized regression coefficients β in the measurement models of EE, SI, CC, WA, and AB are all located at [−1,1], and the parameters estimated by the model are reasonable. The explanatory rate of PE three endogenous variables, PE1(93.3%) > PE2 (69.3%) > PE3(65.8%), with strong explanatory force. Similarly, according to the square *R*^2^ value of the multivariate correlation coefficient, under the influence strength:

EE3(55.0%) > EE1(54.9%) > EE4(49.4%) > EE2(42.6%),

SI3(88.0%) > SI1(73.4%) > SI4(63.6%) > SI2(47.4%),

CC1(82.9%) > CC4(75.6%) > CC3(64.4%) > CC2(62.4%),

WA2(71.1%) > WA1(49.5%) > WA3(39.8%),

AB3(69.6%) > AB2(56.6%) > AB1(54.8%).

### 4.4 Mediating effect test

Currently, the academic circle uses the product of Bootstrap direct test coefficient to test the mediation effect. This study tested the mediating effect using Bootstrap in Amos 22.0. Two thousand samples were set. Set the confidence level to 95% in both bias-corrected confidence intervals and percentile confidence intervals in Table 3.

**Table 3:** Bootstrap analysis of mediating effect.

Model path	Standardized effect value β	95% bias-corrected confidence intervals			95% percentile confidence intervals			Hypothesis testing
		Lower	Upper	*p* value	Lower	Upper	*p* value	
PE → WA → AB	0.435	0.303	0.604	**(0.001)	0.296	0.595	**(0.001)	Pass
EE → WA → AB	0.389	0.184	0.630	**(0.001)	0.181	0.617	**(0.001)	Pass
SI → WA → AB	0.438	0.298	0.596	**(0.001)	0.292	0.593	**(0.001)	Pass
CC → WA → AB	0.121	0.037	0.212	**(0.005)	0.029	0.202	*(0.011)	Pass

*p < 0.05; **p < 0.01; ***p < 0.001.

Table 3 shows that in the intermediate path PE → WA → AB, the standardized indirect effect coefficient is 0.435, 95% bias-corrected confidence intervals are between low and high [0.303,0.604],95% percentile confidence intervals are between low and high [0.296,0.595], both excluding 0 and *p* < 0.001, indicating the mediating effect of WA in the process of PE on AB. Similarly, other mediating effects have been shown.

### 4.5 Moderating effect test

This study used the Process plug-in (Macro Model 1) of SPSS to analyze the simple adjustment effect and the Johnson-Neyman (J-N) analysis method to observe the change of the path effect value of “WA → AB” with the change of the value of PT. Regression analysis results show that PT has a significant positive moderating effect on the “WA → AB.” The non-normalization coefficient of the interaction between WA and AB is 0.0382(*p < 0.05), the 95%CI range is 0.0128 ~ 0.4523, and the confidence interval does not contain 0. Hypothesis H6 is verified. The global path model’s coefficient of determination (R^2^) under the action of adjustment variables is 0.5733.

**Figure 4:**
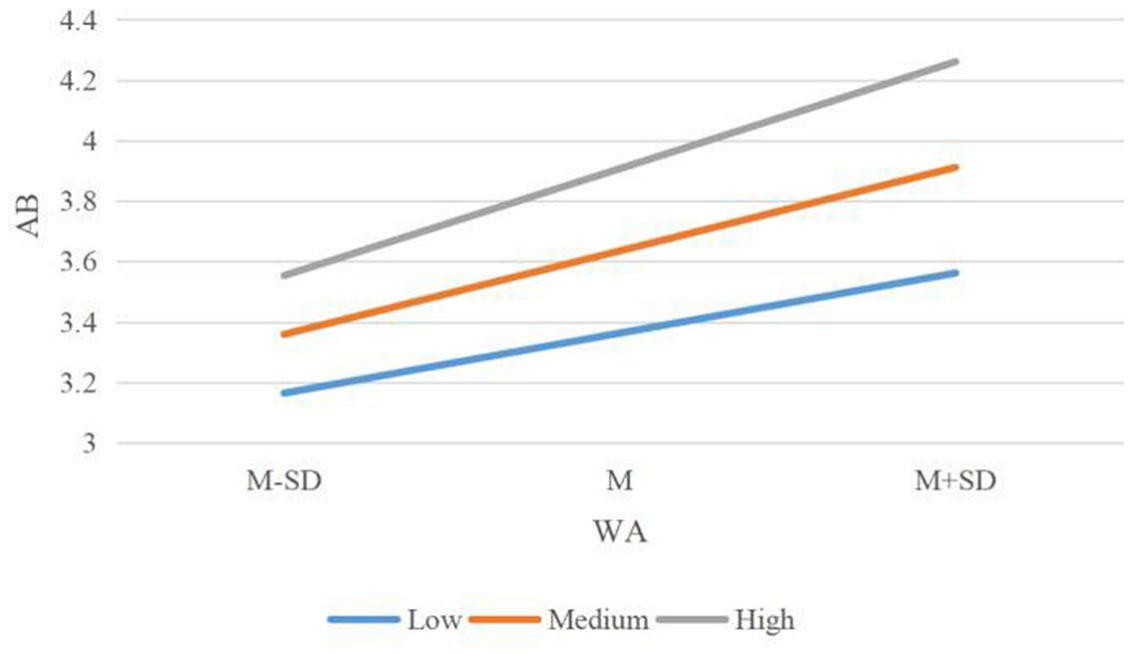
The moderating effect of PT on “WA → AB” relationship.

To reflect the moderating effect of PT more clearly, a simple slope diagram of WA on AB in the high (Mean + SD), medium (Mean), and low (Mean-SD) PT groups were obtained (Figure 4). When PT of construction workers is low, WA has a significant positive predictive effect on AB (*p* < 0.001); When PT was higher, the predictive effect was still significant, and the predictive power increased gradually (p < 0.001). Practical actions taken by construction workers are best when both PT and WA are high. According to the results of the J-N analysis of the adjustment effect, when the PT value is greater than 3.3835, the moderating effect is completely significant, the positive explanatory power of PT on AB increases with the increase of WA, and the positive effect value of WA on AB increases by 0.1916 for every 1 unit increase of PT.

## 5 Discussion

Through an in-depth understanding of MST interactive learning, scene simulation, and practical experience, PT is extended to UTAUT, promoting the application of UTAUT in the architecture, construction, and engineering industries. Based on the extended UTAUT, this study systematically analyzes the influencing factors of WAMST for construction workers and reveals the mechanism of their behavior. This study combines qualitative analysis with quantitative research. It conducts SEM calculation and regression analysis based on a literature review. The results indicate the direction for improving the safety production capacity of construction workers.

### 5.1 Adopt supportive policies, enhance the capability of penetrating supervision

For the government, this study discusses the significant impact of SI. It points out the role of government policy introduction and regulatory capacity in developing MST. Relevant research provides suggestions for the government to command and command the overall situation actively. It provides ideas for promoting the realization of safe production in construction projects.

As shown in Figure 3, SI has the most significant positive effect on WA. The path coefficient of influence of SI1 policy orientation on SI standardization is 0.857, and the measurement model shows a significant influence. Therefore, through policy guidance, the government can improve the application rate of MST in construction enterprises and the enthusiasm of construction workers in safety management (53). For example, establishing government, enterprise, and social diversification of construction workers in the universe of ST funds investment mode reduces the cost of project safety management (12, 54). Regularly or randomly check the project safety management situation and timely report to the superior department. They linked project safety management with performance, evaluation, and other economic benefits, strengthening production safety supervision from positive and reverse incentives (55, 56).

However, basic methods can only show the best implementation results in a particular range. Designing and developing a multidimensional intelligent collaborative management platform with construction workers’ dynamic data management is necessary (57, 58). Through the underlying environment, platform support, core database, analysis algorithm, and platform application, the application terminal of five levels is applied, the innovation policy system is open, the consultation is released, the information update, the data monitoring, the performance system, and the recognition system. In this way, the regulatory authorities can dynamically grasp the relevant information of the construction workers participating in MST to realize all-round and whole-process penetrating supervision. It effectively breaks the data of the layers of embedded data, breaks the boundary of the Internet, controls the construction project’s actions and the false reporting, and implements adequate supervision of the whole process.

### 5.2 Establish a reasonable reward and punishment mechanism, create a safe atmosphere for the project

The empirical results show that PE significantly affects the WAMST. PE represents the perceived usefulness of construction workers to the MST. At present, construction workers need better professional skills and better safety awareness, which is not conducive to the professional ability training and safety management of construction teams (59). MST is more results-oriented for construction workers, focusing on reducing costs, getting rewards, and self-improvement (60). Therefore, the intelligent collaborative terminal of “Web+APP+ WeChat public account + team robot” can train construction workers on risk tips, operation points, and emergency management. The intelligent collaborative terminal will transmit the safety production requirements to the first-line construction personnel and support the high-quality development in the construction industry. For example, construction workers can learn ST courses and answer questions to earn points by designing a points system platform (61, 62). Using study points can redeem for different supplies, including mineral water, energy drinks, bread, instant noodles, ham sausages, and cigarettes. The project adopts the race method for integral management, real-time ranking of critical safety data, and automatic information sending. Moreover, it organizes ST competitions, calculates and ranks the learning points of individuals and groups, and rewards the top individuals and groups with cash, ranging from 300 yuan to 500 yuan for individuals and 1,000 yuan to 3,000 yuan for groups. The lower-ranking group deferred payment of about 20% of the project measurement money.

According to the standardization of SI2, SI3, and SI4, the standardized influence path coefficient, the construction workers’ acceptance will be influenced by their own, the workers, and the group leader. It shows that in the construction industry, the security atmosphere is closely related to the internal working environment and individual security status of the project, and a good security atmosphere can improve the acceptance will of the construction workers and reduce the probability of the whole trip. On the one hand, the project safety manager and the group leader increase the supervision and construction workers to strengthen the supervision and encourage the construction workers to actively use the MST and the incentive mechanism is encouraged to ensure the safety of the builders and enhance the overall safety atmosphere. On the other hand, project security managers focus on communication and feedback with construction workers and team leaders (63). Establish a suitable communication mechanism between the group and the project to ensure the safety of information interaction. In the course of the class, the group robot combines artificial intelligence technology, automatically identifies the group information through face recognition, automatically codes to upload to the cloud, and forms the management desk of the class, which can meet the requirements of the project safety manager multidimensional query through the intelligent storage. The project manager communicates with the group members according to the feedback information and resolves the safety problem quickly. Group members can also provide feedback to the safety manager through weekly meetings, feedback boxes, investigations, WeChat, and interviews.

For the project, this study points out that a good safety atmosphere, as an MST incentive tool, has a significant advantage in improving the WA of construction workers. The design idea of the reward and punishment mechanism of the project is also provided. It will help project managers organize construction workers to complete ST and improve both sides’ communication and feedback efficiency.

### 5.3 Excavate the characteristics of construction workers, strengthen the level of safety cognition

According to SEM results, CC and EE have a certain degree of influence on the WAMST for construction workers. CC refers to the differences in evaluating construction workers on the environment, equipment and facility guarantee, content understanding, and ease of operation in the MST. Due to the differences in education, age, family conditions, working years, and other aspects, construction workers’ safety cognition situation is different. Therefore, the construction workers’ education level, age, economic status, work experience, and other information should be considered in the formation of the team, and the construction workers should be reasonably matched to improve the WAMST and the effect of ST and improve the efficiency of safety management.

EE refers to the degree of sensory stimulation, virtual interaction, immersive experience, and perceived pleasure of construction workers in the MST. The diversified interactive ways of virtual space, the authenticity of the expression, and the on-the-spot experience can immerse the construction workers. The immersive experience shows the costs and losses of unsafe behavior and can promote positive training attitudes among construction workers. ST in construction takes the form of meetings, which are often dull for construction workers and do not provide immediate results (64). MST provides some fun for construction workers and breaks the limitation of the unilateral output of traditional ST. Visual safety disclosure and comprehensive linkage experience enable construction workers to have particular control abilities and make understanding and accepting safety knowledge easier. Through data mining, data processing, and personalized analysis, a data computing center is set up to give ST learning content accurately to construction workers, helping front-line operators of various construction teams intuitively understand the theoretical knowledge of ST. It helps construction workers absorb advanced technology and enrich their safety cognition.

### 5.4 Create ubiquitous intelligent learning space, achieve accurate services

According to the regression analysis results, PT has a significant regulating effect on the “WA → AB” path and the positive explanatory power increases with the increase of WA. Generally speaking, MST is a new technology, and construction workers have anxiety and distrust about its professionalism, authenticity, progressive nature, privacy, and service. In order to solve the existing problems, the MST knowledge base should be built based on investigation, concept recognition, case analysis, and data mining (65). Based on the characteristics of digital twins, such as the entire cycle, fundamental factor, whole space, and complete data, intelligent and inclusive ubiquitous intelligent learning space is created under the cooperation of multi-dimensional intelligent terminals to provide integrated, situational, and interactive learning modes. These practices supplement the actual content of the MST and will play an essential role in future construction engineering practices.

In addition, this study constructed an index system including PE, EE, SI, CC, PT, WA, and AB. It explored a complete framework of factors influencing construction workers’ WAMST. Previous studies on the ST of construction workers are mostly thinking in the traditional mode, and there is a lack of systematic discussion on the application of artificial intelligence, digital twins, blockchain, the Internet of things, and other technologies in the ST of construction workers (66). This framework significantly contributes to the willingness of construction workers to participate and helps improve safety awareness. Combined with the learning characteristics of construction workers, the ST points are accurately translated into situational and immersive 3D animation through digital twin technology, which can highly restore the construction points of roadbed engineering, pavement engineering, tunnel engineering, bridge engineering, housing engineering, and other construction points, and demonstrate the easy-to-understand safety knowledge (67). Establishing a data computing center with the core of “data mining + data processing + personalized analysis” is helpful to realize the accurate push.

After explaining the results, some limitations should be noted.

Regarding research content, this study only explores the factors influencing the WAMST for construction workers. In the future, more empirical studies are needed to explore the mutual relationship and interaction among factors. At the same time, according to the current development status of the metaverse, we should discuss the performance and implementation effect from the MST perspective. Considering individual characteristics, the intergenerational, educational, and stage differences in WAMST are also worth further consideration.

Regarding research methods, this study uses the combination of UTAUT and SEM for empirical analysis. UTAUT ensures the logic and rigor of the study to a certain extent, and SEM has advantages in the description of multivariate relations. However, the causal relationship between variables expressed by SEM depends on cross-sectional data, which can be discussed with DEMATEL and the interpretive structural model. ST for construction workers is a complex and dynamic process. It is difficult to predict the changing trend of the WAMST in the future utilizing SEM. Compared with traditional quantitative methods, system dynamics not only take all the influencing factors into account, but its sustainable systematic and forward-looking characteristics are more suitable for evaluating the WAMST for construction workers.

Regarding the research perspective, MST is still in the early stage of development. With the improvement of metaverse scenarios, the team leader and project safety managers should be considered. Therefore, we will analyze the differences in the WAMST for team leaders and project safety managers and explore the reasons for the differences.

## 6 Conclusion

Based on UTAUT, this study focuses on the WAMST for construction workers in front-line operations. It discusses the influence path and internal mechanism through SEM and regression analysis, which makes up for the limitations of subjective factors to a certain extent. This study proves that UTAUT suits the WAMST for construction workers. PE, EE, SI, and CC have a significant positive effect on WA, and WA has a significant positive effect on AB. Considering the MST characteristics, PT was introduced to UTAUT, which had a significant positive moderating effect on WA → AB, providing a new analytical framework for subsequent studies. The observed variables of PE, EE, SI, CC, PT, WA, and AB were analyzed in depth to reveal the internal interaction among factors. The results guide developing governments, enterprises, projects, and construction workers. For example, the government should adopt supportive policies to enhance the capability of penetrating supervision. Creating ubiquitous intelligent learning spaces and achieving accurate services are essential for enterprises. For the project, it is necessary to establish a reasonable reward and punishment mechanism and create a safe atmosphere. This framework significantly contributes to the willingness of construction workers to participate and helps improve safety awareness. Although the conclusions presented are compelling, they should be challenged and validated by future studies.

## Data availability statement

The raw data supporting the conclusions of this article will be made available by the authors, without undue reservation.

## Ethics statement

Ethical review and approval was not required for the study on human participants in accordance with the local legislation and institutional requirements. Written informed consent from the participants was not required to participate in this study in accordance with the national legislation and the institutional requirements.

## Author contributions

ZG: Conceptualization, Data curation, Formal analysis, Writing – original draft. QW: Conceptualization, Methodology, Supervision, Writing – original draft. CP: Data curation, Methodology, Writing – review & editing. SZ: Data curation, Investigation, Writing – review & editing. BY: Data curation, Resources, Writing – review & editing.
